# Co-morbidity and systemic inflammation as drivers of cognitive decline: new experimental models adopting a broader paradigm in dementia research

**DOI:** 10.1186/s13195-015-0117-2

**Published:** 2015-03-24

**Authors:** Colm Cunningham, Edel Hennessy

**Affiliations:** Trinity College Institute of Neuroscience and School of Biochemistry and Immunology, Trinity College Dublin, Dublin 2, Republic of Ireland

## Abstract

Dementia prevalence increases with age and Alzheimer’s disease (AD) accounts for up to 75% of cases. However, significant variability and overlap exists in the extent of amyloid-β and Tau pathology in AD and non-demented populations and it is clear that other factors must influence progression of cognitive decline, perhaps independent of effects on amyloid pathology. Coupled with the failure of amyloid-clearing strategies to provide benefits for AD patients, it seems necessary to broaden the paradigm in dementia research beyond amyloid deposition and clearance. Evidence has emerged from alternative animal model approaches as well as clinical and population epidemiological studies that co-morbidities contribute significantly to neurodegeneration/cognitive decline and systemic inflammation has been a strong common theme in these approaches. We hypothesise, and discuss in this review, that a disproportionate inflammatory response to infection, injury or chronic peripheral disease is a key determinant of cognitive decline. We propose that detailed study of alternative models, which encompass acute and chronic systemic inflammatory co-morbidities, is an important priority for the field and we examine the cognitive consequences of several of these alternative experimental approaches. Experimental models of severe sepsis in normal animals or moderate acute systemic inflammation in animals with existing neurodegenerative pathology have uncovered roles for inflammatory mediators interleukin-1β, tumour necrosis factor-α, inducible nitric oxide synthase, complement, prostaglandins and NADPH oxidase in inflammation-induced cognitive dysfunction and neuronal death. Moreover, microglia are primed by existing neurodegenerative pathology to produce exaggerated responses to subsequent stimulation with bacterial lipopolysaccharide or other inflammatory stimuli and these insults drive acute dysfunction and negatively affect disease trajectory. Chronic co-morbidities, such as arthritis, atherosclerosis, obesity and diabetes, are risk factors for subsequent dementia and those with high inflammatory status are particularly at risk. Models of chronic co-morbidities, and indeed low grade systemic inflammation in the absence of specific pathology, indicate that interleukin-1β, tumour necrosis factor-α and other inflammatory mediators drive insulin resistance, hypothalamic dysfunction, impaired neurogenesis and cognitive function and impact on functional decline. Detailed study of these pathways will uncover important mechanisms of peripheral inflammation-driven cognitive decline and are already driving clinical initiatives to mitigate AD progression through minimising systemic inflammation.

## Introduction: beyond amyloid beta

### Poor association between amyloid and cognitive decline

Dementia causes loss of memory function and altered behaviour and gradually destroys functional abilities and independence. Its prevalence increases sharply with age and Alzheimer’s disease (AD) apparently accounts for more than 75% of cases. It is increasingly clear, however, that amyloid beta (Aβ) and Tau pathology cannot account for all AD patients: a large proportion of non-demented individuals in the population have significant Aβ and Tau pathology without any signs of dementia [1] and a rather small proportion of dementia risk is attributable to amyloid pathology at death [2]. That is to say that patients with a clinical diagnosis of AD often show a spectrum of pathology encompassing features of vascular, classical AD and other neuropathologies rather than ‘pure AD’. Despite this, the vast majority of research in the AD field has focussed on the build-up of Aβ, but recent clinical trials with amyloid-lowering strategies, including active and passive vaccines and γ-secretase inhibitors, revealed no significant improvement in cognitive or functional outcomes even in mild to moderate AD patients. Those active immunization cases that have come to *post mortem* have shown that all patients die with late-stage dementia, regardless of the success of amyloid removal [3]. These data suggest that other avenues to slowing progression must be explored. Furthermore, given that the vast majority of AD cases (that is, late onset AD) do not carry mutations in the genes (*APP*, *PS1*, *Tau*) on which the amyloid transgenic mouse models have been based, it is clear that alternative animal model systems to study cognitive decline are also required to complement these amyloid transgenic studies.

### The innate immune response is important in dementia

Over the past decade, genome-wide association studies have revealed a large number of common variants that are associated with a small increased risk of AD, including several genes involved in innate immunity, such as *CLU*, *CR1*, *PICALM* [4] and *SIGLEC3* (*CD33*) [5]. In addition there are loci of much more significant risk, such as *TREM2*, a macrophage gene involved in phagocytosis and suppression of pro-inflammatory phenotype in microglia [6]. These AD loci all suggest altered macrophage phagocytic function. However, it is important to stress that altered macrophage function may occur anywhere in the body and these polymorphisms do not specifically predict altered microglial function: they predict differential peripheral macrophage responses too. Individuals taking non-steroidal anti-inflammatory drugs (NSAIDs) during middle age are significantly protected from subsequent development of AD [7] and it may be instructive to recall that these medications were taken to treat peripheral inflammatory conditions like rheumatoid arthritis (RA). The possibility that their protective effects against AD are mediated in the periphery has been little discussed. The growing number of macrophage genes implicated in AD and other neurodegenerative diseases might be collectively conceptualised as reflecting the importance of the proportionate innate immune response to pathological change occurring anywhere in the body: overzealous responses might be damaging but insufficient responses might also be detrimental to the tissue. A recent study, which analysed patients with high amyloid but no dementia, showed a less inflammatory microglial response to the tissue amyloid than those high amyloid patients that did develop dementia [8]. Thus, a proportionate response to amyloidosis may even be more important than the amyloidosis itself in determining the consequences for brain function.

### Medical illness and inflammation are associated with cognitive decline

Systemic inflammation is emerging as a significant driver of cognitive decline in the aged and vulnerable brain. Clinical epidemiology studies of multiple co-morbidities reveal contributions to cognitive decline: obesity, diabetes and atherosclerosis have inflammatory components and these conditions increase the risk of AD. Significantly, the individual’s inflammatory status appears to be a key driver of this risk [9]. Acute medical illness also appears to have robust impacts. Delirium is an acute neuropsychiatric syndrome triggered by various medical illnesses and it has become clear that these acute episodes also predict long-term cognitive decline [10]. Importantly, this more rapid cognitive decline can be dissociated from amyloid levels: in a longitudinal study of aging (Vantaa 85+) episodes of delirium increased the risk of dementia by eight-fold, but while dementia in the overall population was strongly associated with Aβ plaques, Tau tangles, infarcts and α-synuclein Lewy bodies, those associations were lost in patients that became demented after delirium [11]. The prediction arising from this is that how the body responds to medical illness or trauma has significant impacts on the integrity of the brain and may accelerate decline in function in these individuals in ways that are independent of Aβ. Synaptic loss is a stronger correlate of cognitive decline than Aβ plaques or Tau tangles [12] and, although not part of the Consortium to Establish a Register for Alzheimer’s Disease neuropathology assessment, these changes in neuronal integrity hold the key to the loss of function that distinguishes the demented from the merely amyloid-positive.

In the current article we will briefly review clinical evidence for the role of peripheral inflammatory insults/conditions on progression of cognitive decline and will examine the basic research approaches to understanding the contribution of such influences to neurodegeneration. A key focus will be to emphasise that systemic inflammation and co-morbidity can significantly influence cognitive decline in animals without mutations in *APP* and *Tau* genes, thus focussing on research oriented towards late onset dementia. Therefore, although we will discuss Alzheimer’s transgenic studies where relevant, we will give less attention to these than to alternative model systems.

## Acute systemic inflammation

### Severe sepsis causes significant brain injury

**Figure 1:**
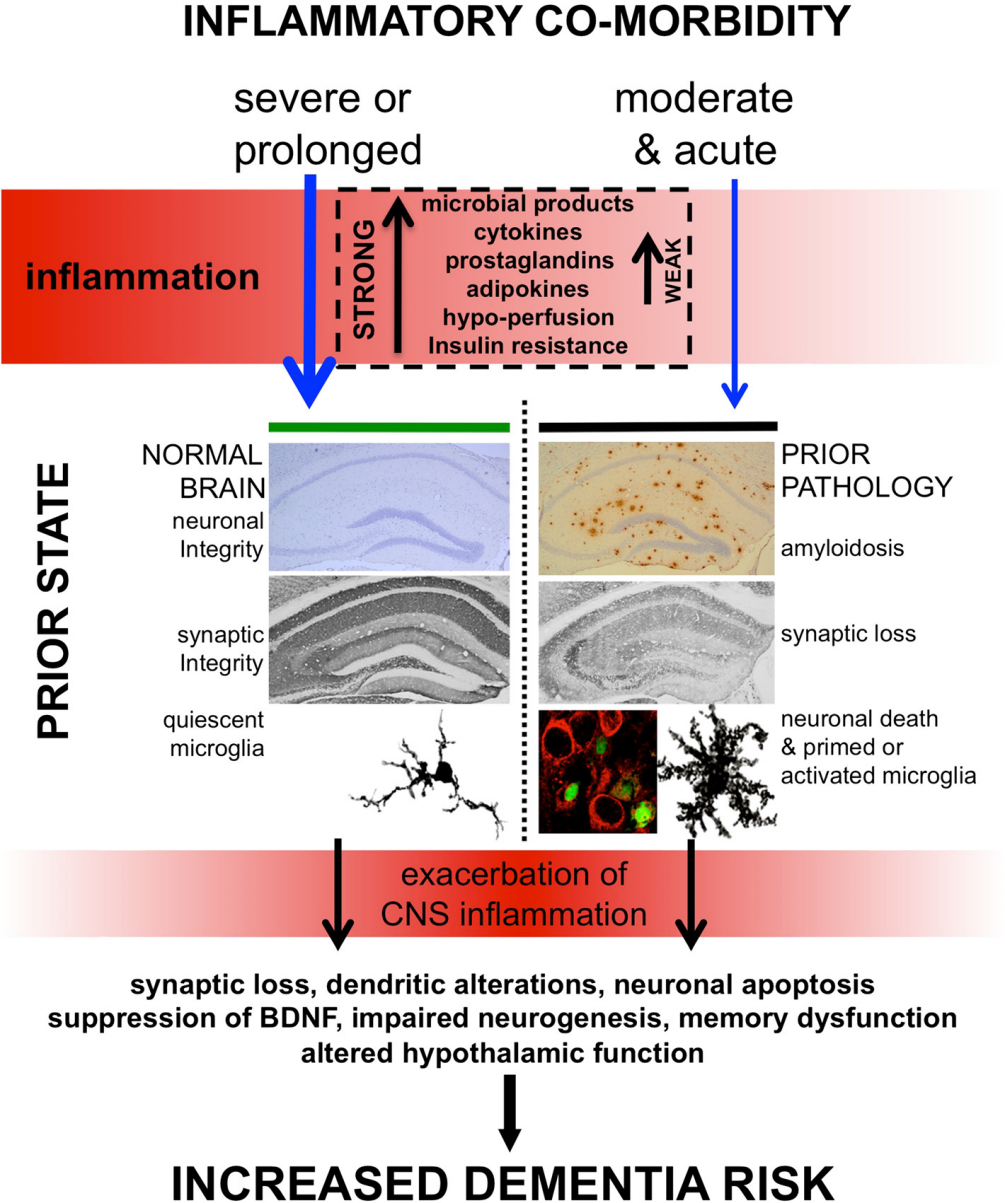
**Inflammatory co-morbidities damage the brain.** Severe (that is, severe sepsis) or prolonged systemic inflammation (that is, diabetes, atherosclerosis, obesity, arthritis), even when superimposed on the normal healthy brain (left: intact synaptic integrity and normal ramified microglia shown), can activate microglia and contribute to changes deleterious for cognitive function and thus increase dementia risk. Strength of induction of inflammatory mediators is shown in the dashed box and echoed by the red gradient. Similarly, when superimposed upon the already pathological brain (right: comprising β-amyloidosis, synaptic loss, neuronal death (green apoptotic nuclei in red-labelled neurons) and microglial activation), even relatively mild/moderate acute systemic inflammation can switch the phenotype of primed microglial cells to produce robust exacerbation of central nervous system (CNS) inflammation and to produce damage in the brain, which can contribute to long-term cognitive decline. Severe or prolonged inflammation superimposed on the already pathological brain is predicted to have even more deleterious consequences for trajectory of decline. Figure adapted from [106] and used with permission of Cambridge University Press. BDNF, brain-derived neurotrophic factor.

**Figure 2:**
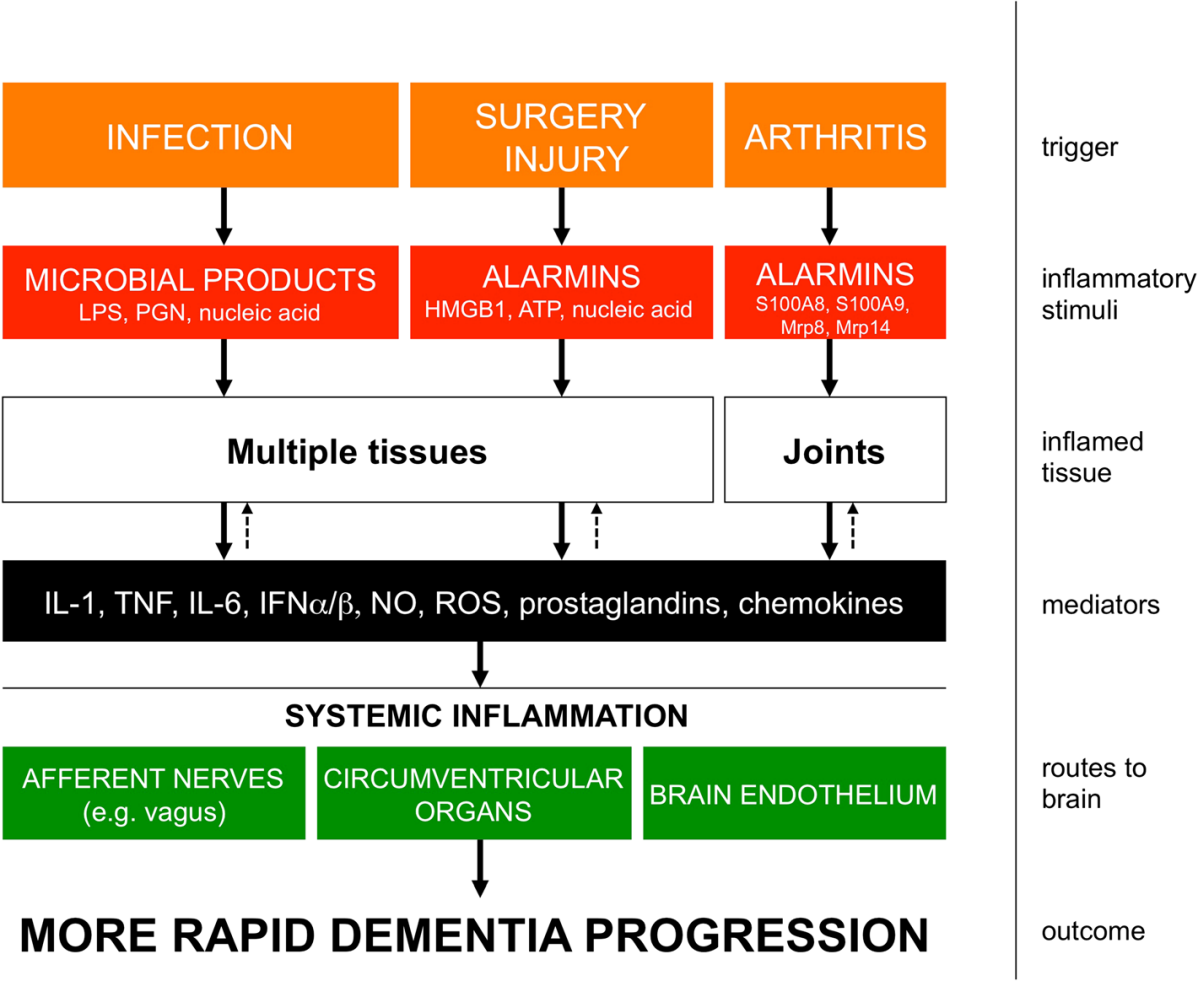
**Recognition of microbial products and alarmins to induce systemic inflammation and impacts on the brain.** Pathogen-associated molecular patterns (PAMPs) and damage-associated molecular patterns (DAMPs or alarmins) induce systemic inflammatory mediators in multiple tissues of the body after infection, surgery, injury or arthritis. Although some aspects of the pathways shown remain unclear, it is clear that all conditions can bring about elevated systemic inflammatory mediators and that these can signal to the brain via well established routes, including direct neural activation via afferent nerves and activation of inflammatory cells in circumventricular organs lacking a patent blood–brain barrier, allowing secretion of inflammatory mediators into the brain parenchyma and activation of soluble mediators at the brain endothelium. Direct impacts on brain pathology or on cognitive function have been shown for all of these insults. Dashed arrows indicate that though these mediators are the result of inflammatory stimulation in the tissues/joints, they also contribute to the ongoing inflammation in those tissues. HMGB1, high mobility group box-1; IFN, interferon; IL, interleukin; LPS, lipopolysaccharide; NO, nitric oxide; PGN, peptidoglycan; ROS, reactive oxygen species; TNF, tumour necrosis factor.

Brain damage resulting from severe sepsis is well known to occur in humans [13] and after ICU-associated delirium up to one-third of patients develop long-term impairments equivalent to traumatic brain injury [14] independent of illness severity [14]. Outcomes are clearly worse contingent on age at admission to the ICU [15] but the resulting inflammation is clearly severe enough to cause significant injury even in young and otherwise healthy individuals (Figure 1). In rodents high-dose bacterial lipopolysaccharide (LPS; 5 to 10 mg/kg), mimicking Gram-negative bacterial infection, induces robust central nervous system (CNS) inflammation, microglial-inducible nitric oxide synthase, neuronal death, blood–brain barrier breakdown and long-term cognitive decline with causative roles described for both inducible nitric oxide synthase and tumour necrosis factor (TNF)-α [13]. LPS acts directly at the brain endothelium but also activates multiple systemic inflammatory mediators and alarmins, which propagate the inflammatory signal throughout the body (Figure 2). Similarly, high mobility group box-1, interleukin (IL)-1β and NADPH oxidase have been shown to have roles in long-term cognitive impairment induced in the cecal ligation and puncture model of polymicrobial sepsis [16-18]. Thus, irrespective of roles in acute cognitive deficits, it seems that inflammation significantly contributes to subsequent neuronal death, denervation and cognitive impairment. Delirium occurs in around half of all ICU patients and patients are more likely to subsequently develop dementia, but delirium and associated brain injury may push patients towards a dementia diagnosis that is not associated with Aβ [11]. Further studies in this domain are likely to reveal molecular mechanisms contributing to cognitive decline in the population.

### Lipopolysaccharide and other acute systemic inflammatory stimuli exacerbate existing disease

The past decade has seen significant interest in the impact of less severe systemic inflammation on the degenerating brain. In a simple conceptual advance [19], our laboratory used a single challenge with bacterial endotoxin, LPS (500 μg/kg intraperitoneally) superimposed upon chronic neurodegeneration induced by prion disease, to demonstrate that the principal brain macrophage population, the microglia, were primed by primary neurodegenerative pathology to produce exaggerated CNS responses to acute systemic inflammation [20] and that this led to acutely increased neuronal cell death [20], accelerated disease progression [21] and acute cognitive dysfunction resembling delirium [22] (Figures 1 and 2). We used the ME7 model of prion disease because it shows progressive synaptic loss, extracellular amyloidosis, microgliosis and robust neuronal loss, which is accompanied by robust behavioural cognitive and neurological decline [21]. While amyloid transgenic models offer excellent opportunities to examine the inflammatory response to amyloid plaques, they do not present robust neurodegeneration and are better regarded, even by their originators, as models of mild cognitive impairment and are less suitable to address interactions between systemic inflammation and existing neurodegeneration. Microglial priming has been confirmed in aged rodents [23], animal models of AD [24], Parkinson’s disease [25] and axonal degeneration [26]. Several molecules, including CCL2, CSF-1, and complement factor C3, are increased in the brain during neurodegeneration and prime microglia, while the loss of microglial inhibiting molecules such as CD200 [27], fractalkine [28] and TREM2 [29] and neurotransmitters such as noradrenaline, acetylcholine and gamma aminobutyric acid may also contribute to the primed state (reviewed in [30,31]). Since these molecules and this cellular state control the CNS amplification of inflammatory signals arriving from the periphery, further elucidation of these pathways will be important in developing strategies to lessen the CNS burden of systemic inflammation.

**Figure 3:**
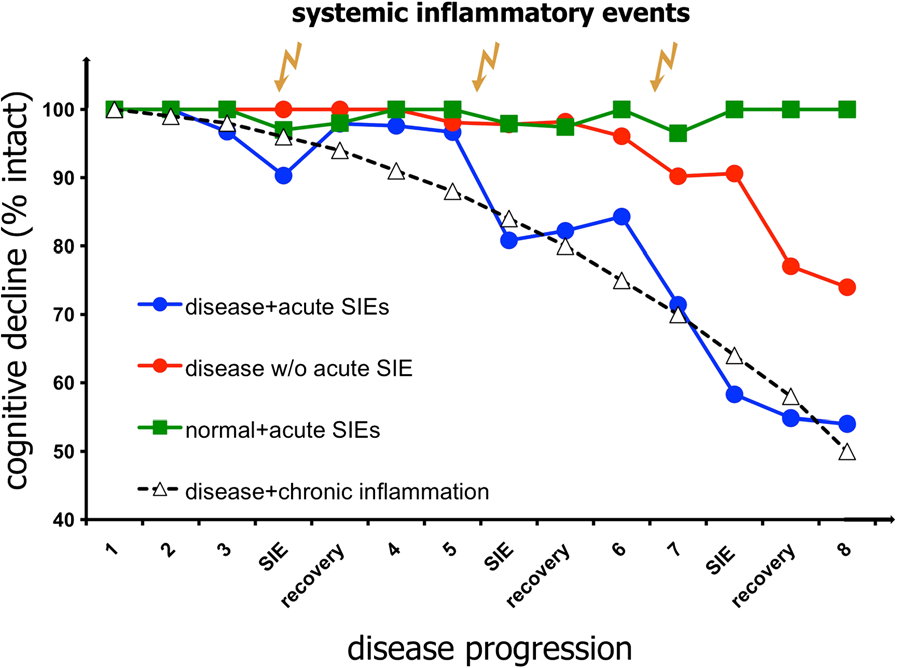
**Altered trajectories.** Cognitive function may decline via stepwise decrements upon a declining baseline due to the cumulative effect of multiple acute systemic inflammatory events (SIEs; shown as lightning strikes, with corresponding acute decrements shown on the blue trajectory) but may also progress more rapidly due to the ongoing effects of chronic inflammatory co-morbidities (black, dashed trajectory) such as those discussed herein. The prediction is that underlying pathology such as amyloid beta may not manifest as dementia, or will manifest significantly later (disease without (w/o) acute SIEs, red trajectory), without the influence of these co-morbid factors (data based on [22,23,45]).

The diseased brain is primed not only to subsequent LPS challenges but also to other inflammatory stimuli: ME7 animals also showed exaggerated CNS IL-1β and also type I interferon (α/β) responses to the synthetic double-stranded RNA poly I:C, which mimics the acute phase response to systemic viral infection. Poly I:C induced both acute and longitudinal exacerbation of chronic neurodegenerative disease [32]. Moreover, three poly I:C challenges, each 2 weeks apart, showed that each successive challenge produced acute onset deficits that were progressively more severe and less reversible as the underlying disease progressed [32] (Figure 3). This mimics the fluctuating and variable rate of decline seen in AD patients [33] and suggests that multiple systemic inflammatory insults contribute, in a cumulative way, to the progression of cognitive decline. A rather different take on this ‘multiple hit’ hypothesis was also advanced in studies that began with systemic poly I:C challenge to wild-type pregnant dams during late gestation [34]. This viral mimetic induced inflammation and increased hippocampal amyloid precursor protein (APP) fragments in the aged offspring and if poly I:C was repeated in adulthood (4 months), these features were strongly exacerbated, inducing amyloid-like plaques despite the lack of human mutated APP in these non-transgenic animals. When poly I:C challenges were made in triple transgenic mice containing mutations in *APP*, *PS1* and *Tau*, inflammation induced APP fragments to act as a seeding point for senile human-like Aβ deposits and drove Tau tangle-like structures in neuronal somata, thus recapitulating two key features of human disease, with systemic inflammation as a driver. These authors propose a model where inflammation-induced alteration of APP cleavage is an early step in pathogenesis of AD and tau mislocalisation occurs as a result of axonopathy and is key to cognitive deficits and one in which the senile amyloid plaque itself is a late feature of disease and largely irrelevant to cognitive dysfunction [35].

There have also been several studies with multiple doses of LPS administered to normal animals and to particular transgenic mice broadly demonstrating increased activity of β- and γ-secretase, intraneuronal APP and extracellular amyloid plaques [36,37]; this increase in intraneuronal APP in the triple transgenic (3xTg) model of AD was TNF-α-dependent [38]. Multiple LPS doses also affect tau hyperphosphorylation and tangle pathology in the 3xTg model in a cyclin-dependent kinase 5 (cdk5)-dependent manner [39]. The dosing regime in these studies was prolonged and it is not clear whether these were intended to mimic multiple systemic infections or chronic peripheral inflammatory disease. Repeated LPS challenge can produce tolerance depending on dose and timing [40] and there is evidence for diminished systemic responses to LPS after three to four doses, while CNS synthesis of IL-1α, TNF-α, IL-6, IL-12 and CCL2 was maintained or even exacerbated in the same animals [41,42]. Thus, multiple systemic LPS challenges may prime microglia despite no longer stimulating systemic inflammation. Given that the repeated LPS approach is now frequently used in AD research, and has deleterious consequences for disease, it is important to characterise the evolving response to multiple consecutive LPS changes. One recent study in an inflammatory model of Parkinson’s disease demonstrated that four LPS challenges (1 mg/kg) result in a transcriptome response that is distinct from that induced by a single LPS challenge, with significant activation of the complement and phagosome systems directly leading to complement factor 3 (C3)-dependent neuronal death [43].

It is also important to briefly address the discussion of beneficial versus detrimental effects of acute inflammatory stimulation since several studies suggest that further activation of microglia using LPS is beneficial in clearing Aβ. While we would argue that activating microglia in this fashion would be deleterious to the brain, irrespective of effects on Aβ, it is possible that some aspects of microglial function may be harnessed for beneficial effects. It was recently shown that monophosphoryl lipid A, a chemically detoxified lipid A moiety derived from *Salmonella minnesota* LPS, induced increased microglial phagocytosis of Aβ without the overt pro-inflammatory responses usually associated with LPS [44]. The outcomes of such additional microglial activation for the brain require study, not only to assess their role in clearance of amyloid but also to assess whether they produce bystander damage during these activities. The successful removal of amyloid plaques by active and passive immunisation strategies did not prove beneficial for patients [3] and the majority of information from the clinical literature would suggest that systemic infection or inflammation leads to worse outcomes in AD patients, including acute delirium and worse long-term cognitive trajectories [10,31].

Finally, although most studies of acute inflammation used LPS to exacerbate underlying CNS disease, other stimuli have been used, including adenovirally mediated systemic expression of IL-1β, active infection, reactivation of latent viruses, ulcerative colitis, periodontal disease, liver injury (bile duct ligation and resection) and indeed chronic stress. Although there is no space to discuss these here, each has their own merits in manipulating aspects of systemic or CNS inflammation to examine the impact on underlying brain pathology (reviewed in [30]).

### Delirium and post-operative cognitive dysfunction

Delirium might be regarded as the clearest evidence that systemic inflammation impacts negatively on the degenerating brain. It is clear that existing cognitive impairment is the biggest risk factor for delirium and, on this background, milder inflammatory insults, including infections, injury and surgery, readily produce the profound acute cognitive, attentional and neuropsychiatric disturbances characteristic of delirium [45]. Patients experiencing delirium have multiple negative outcomes, including long-term cognitive decline, dementia and shortened time to permanent institutionalisation and death [10]. Patients suffering delirium after systemic inflammatory insults such as hip fracture/repair show markedly elevated systemic cytokines [46,47] and inflammatory cytokines are now increasingly demonstrated to be elevated in the cerebrospinal fluid and to be associated with delirium [48,49]. Animal model studies using LPS to mimic acute inflammation are consistent with this, showing causative roles for IL-1β and cyclooxygenase-1-mediated prostaglandins in acute cognitive deficits [50]. Importantly, these changes are only observed in the predisposed brain: whether by the occurrence of microglial priming [20,51], the loss of synaptic connectivity due to progressing disease [52], or loss of the neuromodulatory and anti-inflammatory influence of acetylcholine [53], the diseased brain is vulnerable to the cognitive disrupting effects of systemic inflammation and, after recovery from acute deficits, neurodegenerative disease proceeds more rapidly [21]. It is clear that, at least in the frail brain, surgery also represents a significant inflammatory trauma and many patients suffer post-operative cognitive dysfunction. This was initially attributed to neurotoxic effects of anaesthetics/sedatives but there is an emerging consensus that the primary insult is the inflammatory trauma of surgery in the older patient inducing acute or lasting cognitive deficits. There is evidence that the surgical trauma leads to release of endogenous tissue alarmins such as high mobility group box-1, which act at pattern recognition receptor Toll-like receptor 4 to induce TNF-α and IL-1β, either sequentially or in parallel, and these cytokines can have direct acute effects on cognitive function (Figure 2) [54,55]. With respect to its contribution to long-term decline or dementia, it is worth noting that post-operative cognitive dysfunction does not have a clinical definition and many studies have not been clear on whether acute cognitive dysfunction or a more lasting cognitive decline are interrogated. Most basic research studies use the contextual fear-conditioning paradigm in young healthy rodents, in which conditioning occurs directly before the inflammatory trauma; thus, the task interrogates only dysfunction in memory consolidation at the time of inflammatory trauma. Evidence for lasting dysfunction or indeed decline is thus limited, although there are some reports of longer-term effects and/or neuropathological changes [56]. As such, evidence for roles of IL-1β and TNF-α in surgery-induced contextual fear-conditioning deficits mimic those previously observed after LPS or *Escherichia coli* challenges in the same behavioural paradigm and may be more relevant to acute dysfunction than dementia. Nonetheless, the possibility of important interactions between inflammation and sedation, leading to brain injury, remains an important area to study. Targeting the mechanisms that bring about delirium and/or post-operative cognitive dysfunction may have utility in slowing the progression of dementia.

## Systemic inflammation and clinical Alzheimer’s disease progression: acute or chronic?

There are now many clinical studies indicating that infections and systemic inflammation are associated with clinical AD (reviewed in [57]). Importantly, the impact of acute inflammatory events on cognitive decline has also been prospectively verified in AD patients, demonstrating that carer-reported acute systemic inflammatory events accelerate cognitive decline on the ADAS-Cog scale, and that when these events are accompanied by elevated serum TNF-α this decline was significantly more profound [58]. Notably, there were many patients who showed elevated TNF-α, but whose carers did not report an acute systemic inflammatory event, suggesting that patients with chronic low-grade conditions have elevated systemic TNF-α, and that this impacts on the progression of underlying dementia (Figure 3). This is consistent with a growing animal model literature suggesting that chronic systemic inflammation is a driver of CNS disease, as we discuss below.

## Chronic systemic inflammatory disease

### Arthritis

Epidemiological studies showing that RA patients were protected against the subsequent development of AD led some to suggest that arthritis may actually protect against AD [59]. More recently, a population-based study identified RA as an important risk factor for subsequent dementia generally (risk ratio 2.77) or AD specifically (risk ratio 2.45) [60]. Therefore, it is likely that RA patients take anti-inflammatory treatments for their condition, which in turn protect against the development of AD. Anti-TNF therapies are an effective treatment for RA [61], and recent conference proceedings from the American College of Rheumatology have reported that they significantly reduce the risk of development of AD. This is consistent with prior data demonstrating that the TNF-α level in the serum of AD patients is predictive of accelerated cognitive decline [58]. Although discrete triggers for arthritis remain unclear, multiple studies show that the alarmins S100A8, S100A9, Mrp8 and Mrp14 are released by phagocytes and are present in the synovial fluid, where they activate Toll-like receptor 4 to induce cytokines such as IL-1β and TNF-α (Figure 2), which in turn stimulate further matrix metalloproteinase secretion from chondrocytes [62]. In spite of the epidemiological indications and the robust induction of pro-inflammatory cytokines there are few studies on the interaction between RA and AD using animal models of disease or indeed on the impact of RA on the aged, non-transgenic brain. One recent study reported decreased Aβ, but increased vascular damage and mortality in RA APP/PS1 double transgenic mice [63]. Another study assessed the impact of osteoarthritis on AD pathology in APP/PS1 mice. Since IL-1β is known to contribute to osteoarthritis pathology, the Col1-IL1β^XAT^ Cre inducible model was used to model osteoarthritis and when these animals were crossed with APP/PS1 mice and injected with Cre to induce chronically elevated IL-1β expression, there were significant exacerbations of Aβ deposition and associated microglial activation [64]. No one, to our knowledge, has assessed its impact on cognitive decline and other features of neuropathology and this should be investigated.

### Metabolic syndrome

**Figure 4:**
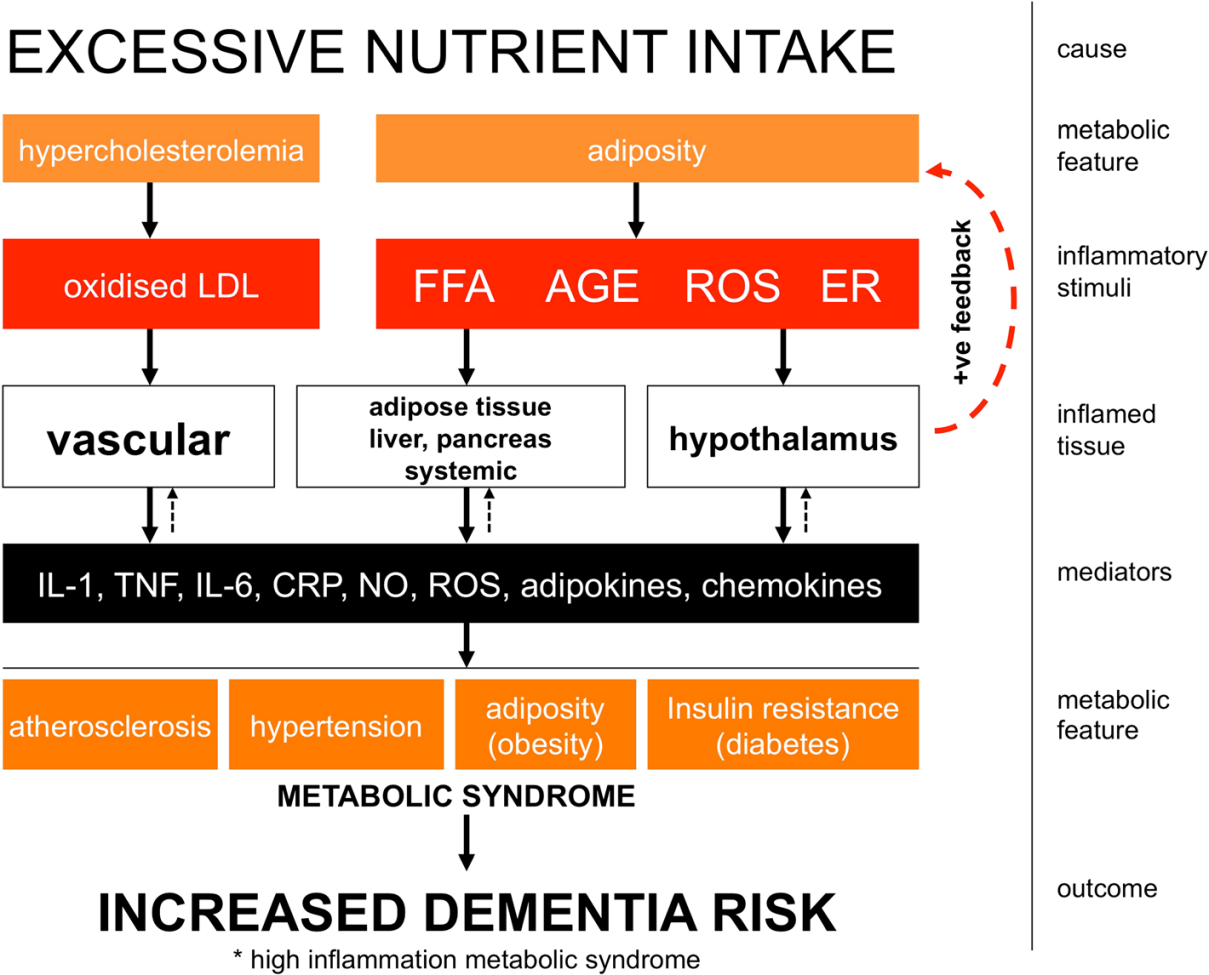
**Inflammatory metabolic syndrome.** This schematic summarises the key inflammatory stimuli arising from excessive nutrient intake, the main tissues experiencing inflammatory changes, the predominant inflammatory mediator output of these tissues and the impact of these changes on propagation of the metabolic syndrome and associated risk for Alzheimer’s disease. In particular it has emerged that hypothalamic inflammation produces hypothalamic dysfunction, which further disrupts central nervous system regulation of appetite and energy expenditure. Dashed arrows indicate that though these mediators are the result of inflammatory stimulation in the tissues/joints, they also contribute to the ongoing inflammation in those tissues. AGE, advanced glycation end products; CRP, C reactive protein; ER, endoplasmic reticulum stress; FFA, free fatty acids; IL, interleukin; LDL, low density lipoprotein; NO, nitric oxide; ROS, reactive oxygen species; tumour necrosis factor.

Obesity, diabetes and atherosclerosis fall under the umbrella of metabolic syndrome (Figure 4), which is the name given to the grouping of at least three of the following features; abdominal obesity, hypertension, hyperglycaemia, hypertriglyceridaemia and low levels of high-density lipoprotein. Metabolic syndrome is a significant risk factor for development of AD but this association was limited to those metabolic syndrome cases with elevated serum pro-inflammatory markers [9], indicating that inflammatory processes associated with, or even underpinning, metabolic syndrome may contribute to dementia progression. Here we briefly review the impact of these co-morbidities on brain ageing in animal models and examine possible inflammatory mechanisms (summarised in Figure 4), while recognising that non-inflammatory mechanisms may also be important.

#### Atherosclerosis

A meta-analysis of epidemiological studies showed a correlation between mid-life serum cholesterol levels and dementia [65]. Atherosclerosis is characterised by elevated low density lipoprotein (LDL; Figure 4), which becomes oxidised and activates macrophages via the scavenger receptor CD36, producing IL-1β via the NLRP3 inflammasome [66,67]. This leads to a state of chronic vascular and systemic inflammation [68]. The acute reactant C reactive protein is most readily measureable and it has been shown that high levels of it are associated with increased microglial activation in human positron emission tomography imaging studies [69].

There are numerous rodent models combining atherosclerosis and AD risk factors in an effort to discern common aetiologies. The addition of a high cholesterol (atherogenic) diet leads to alterations in APP processing and exacerbated spatial learning impairment in the Tg2576 human APP-overexpressing mouse [70]. Apolipoprotein E (ApoE) is a lipid binding protein integral to the metabolism of cholesterol via low density lipoprotein receptor (LDLR) and the Apoε4 allelle is a major risk factor for both atherosclerosis and AD. Removing or overexpressing LDLR modulates cholesterol up or down and can increase or decrease Aβ respectively, suggesting that cholesterol has direct effects on amyloid deposition and/or clearance. Expression of *Apoε4* versus *Apoε3* in mice resulted in impairments in spatial and avoidance memory [71,72]. ApoE-deficient animals (which show a similar phenotype to *Apoε4* allele carrying mice) show elevated inflammation and gliosis associated with their deficient phagocytosis of apoptotic bodies [73] and APP23 mice negative for ApoE fed an atherogenic diet also showed increased endothelial activation and increased vascular pro-inflammatory markers but no alteration in Aβ deposition [74]. Statins have long been used to regulate peripheral cholesterol and meta-analysis shows that these drugs reduced dementia risk [75]. Statins are now recognised to have anti-inflammatory actions [76] and they significantly enhanced memory and reduced Aβ plaque deposition without altering serum lipid levels in an APP overexpression model [77]. These data indicate atherosclerosis affects cognitive ageing and has a robust inflammatory aetiology but precise pro-inflammatory mechanisms contributing to accelerated cognitive decline and AD risk require elucidation.

#### Obesity and type 2 diabetes

Obesity and the frequently associated complication type 2 diabetes are associated with functional deficits in learning, memory and executive functions and with increased risk of dementia [78,79]. Excessive nutrient intake is key in the genesis of obesity and type 2 diabetes: adipocytes and macrophages in the white adipose tissue respond to molecules such as free fatty acids, advanced glycation end products and reactive oxygen species (Figure 4) with the production of TNF-α, IL-1β, IL-6, CCL2 and adipokines like leptin [80]. The cytokines TNF-α and IL-1β can phosphorylate insulin receptor substrate-1 to induce insulin resistance [81], while the Islet amyloid polypeptide deposited in the pancreas can activate the NLRP3 (Nod-like receptor family, Pyrin domain containing 3) inflammasome to drive IL-1β secretion [67,82]. Thus, inflammation has key aetiological roles in obesity and diabetes.

Exposure to a high fat diet (HFD) can induce both obesity and a diabetic (insulin resistant) state in rodents, which means that models of obesity and diabetes are highly overlapping: consumption of a HFD or use of leptin deficient (*ob/ob*) or leptin receptor deficient (*db/db*) mice, which do not respond appropriately to this satiety hormone, have recently been used to examine CNS effects. Consumption of a HFD in normal mice increases hippocampal pro-inflammatory markers IBA-1, TNF-α and glial fibrillary acidic protein, reduces brain-derived neurotrophic factor and dendritic complexity and decreases long-term potentiation, learning ability and impaired working and spatial memory (reviewed in [78]). Chronic HFD also exacerbated peripheral and brain inflammatory responses to LPS [83], indicating a priming of macrophage and/or microglial cells. When superimposed on the ageing brain, HFDs exacerbated systemic inflammation, blood–brain barrier disruption, oxidative damage, hippocampal micro-vascular rarefaction and hippocampal-dependent cognitive decline [84-86]. Alzheimer transgenic models fed a HFD show exacerbated memory impairment as well as increased levels of Aβ oligomers and deposition [87,88]. A HFD in the 3xTg AD model induced memory deficits and exacerbated neuro-inflammation, but these effects were independent of alterations in Aβ or Tau pathology [89].

The obese/diabetic leptin receptor deficient *db/db* mouse exhibits synaptic dysfunction, microglial priming and impaired spatial and object recognition memory. Crossing of APP transgenics with *db/db* mice resulted in increased inflammation, amyloid angiopathy, increased brain atrophy, cortical Tau pathology and exacerbated cognitive deficits but no additional effect on Aβ deposition [90-92]. Insulin resistance in this model also chronically elevates corticosterone, which, like chronic stress [93], contributes to microglial priming, increasing brain IL-1 and TNF responses [94]. Intrahippocampal administration of IL-1 receptor antagonist was protective against obesity-induced neurophysiological dysfunction, indicating that leptin deficiency, via promotion of a pro-inflammatory environment in the brain, may therefore contribute directly to cognitive decline [95].

Use of glucagon like peptide 1, which stimulates insulin, can reverse deleterious effects of HFD on learning and memory, CA1 long-term potentiation and hippocampal glial fibrillary acidic protein, mammalian target of rapamycin and vascular endothelial growth factor [96] and this is now a promising therapeutic target for AD [97]. There are many ways in which reduced efficacy of the insulin receptor pathway may contribute to AD-associated changes and the primary aetiological role of inflammatory mediators in driving insulin resistance (Figure 4) places inflammation at the centre of the obesity/diabetes-associated AD risk.

A more recent development is the finding that increased adiposity, altered adipokines and/or inflammatory mediators (but not body weight *per se*) induce microgliosis [98], cytokine secretion [99] and neuronal dysfunction and death in the hypothalamus [100]. The hypothalamus is a key site of action of insulin and leptin and is the CNS regulator of appetite control and energy expenditure. These pathological changes contribute to furthering the metabolic dysfunction and once again underline the key role of inflammation in metabolic syndrome. Perhaps of even more significance, inflammatory signalling in the hypothalamus (IKK-β and NFκB) also drives frailty and decreases neurogenesis, effectively accelerating aging [101]. This places inflammation in the hypothalamus as a key determinant of rates of cognitive and function decline.

## Is mild systemic inflammation enough?

An excellent study of the impact of low-grade inflammation on brain aging was performed using parabiosis, in which aged and young animals are sutured together at the flanks and ultimately share the same circulation [102]. This demonstrated that exposure to the bloodstream of the aged mouse brought about impaired neurogenesis, electrophysiological evidence of impaired memory function and cognitive impairments in the young animals. Interestingly the opposite was true for old mice exposed to the young bloodstream: some recovery is possible when exposed to the young bloodstream. The authors identified a number of inflammatory factors present in the blood of aged rodents and people, and demonstrated that one of these factors, the chemokine eotaxin (CCL11), was capable of producing the same deficits as exposure to blood from aged rodents [102]. These animals had no specific disease state and simply the elevated inflammatory state of aging was sufficient to bring about some cognitive decline. It seems reasonable to conclude that the same milieu superimposed on an already frail brain will have more significant consequences. Another recent study demonstrated that the ablation of *Nlrp3*, a key subunit of the inflammasome complex that regulates IL-1β maturation and secretion, leads to protection against a number of age-related aspects of functional decline. Significantly, the lack of NLRP3-mediated IL-1 release and activity led to improved glucose metabolism, decreased brain innate immune activation, reduced gliosis, improved cognitive function and extended life-span [103]. While this intersects with recent reports that NLRP3 also contributes to amyloidosis and cognitive dysfunction in the APP/PS1 model of AD [104], it is important to recognise that the former study shows an influence of low grade chronic sterile systemic inflammation on brain aging and cognition in a manner that is IL-1-dependent, in the absence of amyloid pathology. Furthermore, age-associated inflammatory activity in the hypothalamus has whole body effects on ageing, including muscle tone, bone mass, neurogenesis and cognitive function [101] and since the hypothalamus is one of the primary brain centres affected by systemic inflammation this adds weight to the idea that systemic inflammation is a key driver of ageing that encompasses not just brain structures obviously relevant to dementia, but to functional decline of the individual. It is striking that mid-life occurrence of these co-morbidities is where the association with dementia lies and patients taking NSAIDs were protected against subsequent AD development. Directly addressing the hypothesis that systemic inflammation is a driver of dementia is an important priority and, fuelled by the association of elevated serum TNF-α with more rapid cognitive decline [58], the STEADI-09 study (Safety and Tolerability of Etanercept in Alzheimer’s Disease) recently showed that peripheral blocking of TNF-α, using the fusion protein TNF-α inhibitor etanercept, stabilised cognitive function in 20 AD patients with respect to progression in placebo-treated AD patients [105].

## Conclusion

A growing body of clinical and preclinical evidence demonstrates that various peripheral inflammatory insults can exacerbate CNS inflammation, produce *de novo* neuropathology and accelerate cognitive and/or functional decline and these are consistent with epidemiological data for risk factors that we have known about for some time. It can now be said that it is a fact, rather than a theory, that chronic co-morbidities and acute systemic inflammatory episodes contribute to the progression of dementia. Further studies are required in non-transgenic models in order to avoid propagating an over-simplification of the relationship between amyloid and neurodegeneration in a disease that, for the vast majority, occurs in old age and is associated with multiple co-morbid conditions. Animal model studies with co-morbid conditions will be important in delineating the precise role(s) of inflammation in the cognitive and degenerative effects of these major risk factors. APP transgenic mice, which model the genetic risk for early onset AD, do not provide the full pathological spectrum of the late onset human disease and it seems likely that these mice would also recapitulate disease more fully if they accumulated co-morbidities or were experimentally manipulated to do so (Figure 1). Moreover, given the clear contribution of co-morbid inflammation to disease progression, it is important that patients with such co-morbidities are not excluded from clinical trials of novel or repurposed drugs for AD. Testing anti-inflammatory drugs in an environment where typical, rather than selected, co-morbidity-free patients are included may reveal the true contribution of inflammation to progression of dementia.

## Note

This article is part of a series on *The impact of acute and chronic medical disorders on accelerated cognitive decline*, edited by Carol Brayne and Daniel Davis. Other articles in this series can be found at http://alres.com/series/medicaldisorders.

## Abbreviations

AD: Alzheimer’s disease
ApoE: apolipoprotein
APP: amyloid precursor protein
Aβ: amyloid beta
CNS: central nervous system
HFD: high fat diet
IL: interleukin
LDLR: low density lipoprotein receptor
LPS: lipopolysaccharide
NSAID: non-steroidal anti-inflammatory drug
RA: rheumatoid arthritis
TNF: tumour necrosis factor
